# Enhancement of the Mechanical Performance of Glass-Fibre-Reinforced Composites through the Infusion Process of a Thermoplastic Recyclable Resin

**DOI:** 10.3390/polym15153160

**Published:** 2023-07-25

**Authors:** Raffaele Ciardiello, Dario Fiumarella, Giovanni Belingardi

**Affiliations:** 1 Department of Mechanical and Aerospace Engineering, Politecnico di Torino, 10129 Torino, Italy; 2 Interdepartmental Centre J-Tech@PoliTO-Advanced Joining Technologies, Politecnico di Torino, 10129 Torino, Italy; dario.fiumarella@polito.it

**Keywords:** composites, mechanical properties, thermoplastic resin, recyclability, infusion process

## Abstract

Mechanical testing of glass-fibre-reinforced composite (GFRP) plates made of twill fabric and a thermoplastic recyclable infusion resin is presented. The considered thermoplastic resin, ELIUM^®^, is made of poly-methylmethacrylate and can be infused with properly tuned vacuum techniques, in the same manner as all liquid resin. Tensile, flexural, and drop-dart impact tests were carried out to assess the mechanical properties of the composites considering different fabrication conditions, such as the different degassing pressure before infusion and three different infusion vacuum pressures. The work reports a methodology to infuse ELIUM resin at a relatively high vacuum pressure of 0.8 bar. X-ray microtomography analysis showed that the produced laminates are free of defects, differently from what was reported in the literature, where void problems related to a vacuum infusion pressure higher than 0.3–0.5 bar were pointed out. Vacuum pressure values influence the mechanical characteristics of the laminate: when higher vacuum pressures are adopted, the mechanical properties of the GFRP laminates are enhanced and higher values of elastic modulus and strength are obtained. On the other hand, degassing the resin before infusion does not influence the mechanical properties of the laminates. A maximum bending and tensile strength of 420 and 305 MPa were reached by using the vacuum infusion of 0.8 bar with an elastic modulus of 18.5 and 20.6 GPa, respectively. The density of the produced laminates increases at higher vacuum infusion pressure up to a maximum value of 1.81 g/cm^3^ with the fibre volume fraction of each laminate.

## 1. Introduction

Fuel consumption and pollutant emission reductions represent an important target for the transportation industry. Weight reduction is still a very effective way to cut fuel consumption and, as a consequence, greenhouse gases. It has been estimated that, over a year, a 7% fuel saving is obtained for every 10% of the weight reduced from the total car weight. Furthermore, lightweight strategies are also pursued to increase battery autonomy. The automotive industry is replacing conventional materials, such as steel and aluminium, with polymeric composite or hybrid composite/metal materials [1,2,3,4] to produce even lighter structures without the need of compensating for any stiffness reduction. Composite materials can be designed using constituents, matrices, and fibres so that they present mechanical characteristics similar to those of metallic materials, even if they are lighter due to the lower density of the used constituents. The structural elements (frame, hood, roof lining, doors and tailgate, bumpers, dashboard, cross member, suspension arms, seat frame, engine support, cross member, battery support trellis, and crash boxes) [3,4,5,6] can be produced using several specific material structures, including composite materials based on bidirectional fibre fabric systems, to ensure high mechanical performance.

Although composite materials represent the best solution for lightweight applications due to their favourable density, there are some open issues related to their environmental impact since there is no unique technique to recover or recycle these materials [7]. Especially in Europe, the European Directive 2000/53/EC [8] states that all car manufacturers must strictly respect the reusability and recyclability standards for their vehicles. A total of 95% of the vehicle’s weight must be made up of recoverable materials, specifically, 85% through the recovery of the material or the reuse of components and 10% through energy recovery.

Pursuing an alternative perspective, a different class of thermoplastic matrixes for composite materials has been studied and developed in recent years due to their re-processability. One promising thermoplastic resin is Elium^®^ thermoplastic by Arkema. Elium thermoplastic is a reactive methylmethacrylate-based fully recyclable resin [9,10,11,12,13,14,15,16,17,18]. At ambient temperature, it is in a liquid state and presents low viscosity. Due to this peculiarity, it can be used to produce a composite with vacuum-assisted resin infusion technology. Thus, composite material components can be manufactured using the same processes developed for thermoset resins, while its enhanced recycling capabilities have been demonstrated by several studies [14,19,20].

Several studies have already been carried out on the mechanical performance of the Elium thermoplastic resin [21,22,23,24]. They showed that the Young’s modulus and flexural and tensile strengths of the Elium composites are comparable with those of epoxy composites, although the Elium composites withstand greater deformations at failure due to their viscoelastic response.

Several authors investigated the impact response of Elium thermoplastic reinforced with fibres of different natures (glass, carbon, UHMWPE) [25,26,27]. All these authors confirmed that the composites made with ELIUM resin present enhanced impact resistance compared to composite materials prepared with epoxy resin. Further, they showed that impact strength, fracture toughness, and energy absorption at the peak point of the ELIUM composites are significantly higher than those of conventional epoxy composites. ELIUM composites reinforced with glass fibres present structural integrity parameters that are 240% higher compared to composites produced with epoxy resin [25]. This enhancement of the structural integrity parameters can be explained by the higher performance of the adopted matrix: the energy before failure is mainly absorbed due to the elastoplastic and ductile properties of the resin. Drop-dart impact testing carried out at different velocities showed a strain sensitivity related to the Elium microstructure, with improved performance at increasing impact velocity. Other studies [28,29,30,31,32] have also been conducted on the join, welding, dumping, and recycled properties and fatigue capability of the Elium^®^ thermoplastic resin.

Although many studies reported the mechanical properties of Elium composites, there is not a unique adopted procedure for the fabrication of these composites. Bhudolia et al. [33] evaluated the effect of single-stage and multi-stage vacuum level infusion on the laminate’s void content. The optimised vacuum levels that they reported [33] involved a vacuum pressure of 0.5 bar on the flow mesh, which was then reduced to 0.4 bar beyond the flow mesh, with a consolidation pressure of 0.33 bar. Further, the mentioned work analysed the variation in the interlaminar shear strength and the void content as a function of the process parameters, but without any assessment of the mechanical response of the material under quasi-static or impact loads. Furthermore, they [33] proved that a void concentration of 0.88% was achieved using a vacuum infusion pressure of 0.5 bar and a consequent consolidation pressure of 0.33 bar. On the other hand, the use of a vacuum infusion pressure of 0.5 bar without a lower pressure consolidation led to a void concentration of about 6%. Bhudolia et al. [33] also observed that the boiling of the Elium resin can be triggered by the relatively high vacuum pressure and the exothermic reaction of the resin. Of course, vacuum pressure values influence the mechanical characteristics of the laminate. Nugroho et al. [34] showed that higher vacuum pressures increase the mechanical properties of GFRP laminates; in particular, higher values of elastic modulus and strength were obtained.

Since different works suggest the use of low vacuum pressure, and higher vacuum pressures can lead to higher mechanical properties [34], a study of the influence of the Elium resin is proposed to assess the influence of this parameter on the mechanical properties of the Elium composite materials, and to show that a configuration that can infuse GFRP at a consolidation pressure of 0.8 bar is achievable. The aim of the work is also to find an optimised methodology to infuse Elium resin at a higher vacuum pressure. Furthermore, the investigation of different degassing pressures was added to the experimental plan because the technical datasheet suggests the use of degassing for thick laminates and increasing the quality of the laminates. Standard three-point bending, tensile, and impact tests with a drop-dart testing machine were performed to assess the influence of the degassing and infusion pressure on the material mechanical performance. Results evidenced that the infusion pressure significantly affects the quasi-static mechanical response, while the degassing pressure does not influence the resin performance. Computed tomography analysis showed that the infusion at different vacuum pressures (0.5, 0.7, and 0.8 bar) led to low values of the void concentration (0.74–1.17%).

## 2. Materials and Methods

### 2.1. Materials

The thermoplastic poly-methylmethacrylate ELIUM^®^ (Arkema, Colombes, France) was used to produce reinforced glass fibre plates with the Vacuum-Assisted Resin Transfer Moulding (VARTM) process. The Elium^®^ resin at room temperature presents a clean and transparent colour and a low viscosity. The resin is a methylmethacrylate-based polymer (Arkema, Colombes, France) that polymerises by hand-mixing water-free benzoyl peroxide (BPO) hardener (Arkema, Colombes, France) with a weight concentration of 3% in weight. Reckendorf et al. [35] studied the chemical properties of the Elium resin. In particular, Fourier Transform InfraRed (FTIR) analysis showed that the main functionalities of Elium resin are the C–O–C bond, C–H bond (bending), and four distinct peaks of CH3 and CH2 bonds (C–H stretching). The properties of the Elium^®^ resin used in this work are summarised in Table 1, which reports the main mechanical properties of the bare resin together with the viscosity and gel time, which were not assessed in the present work. The technical data were provided by the Elium^®^ producer Arkema (Colombes, France). The E-Glass (CrisTex Composite Materials, Blackburn, UK) used for the experimental activity is a twill 2 × 2 (weight 163 g/m^2^). The warp and weft are 12 and 11.5 threads per cm with a tensile strength of 500 and 450 N/cm. The thickness of the glass fabric is 0.13 mm and the diameter of the fibre is approximately 10 µm.

### 2.2. Manufacturing Process

**Figure 1:**
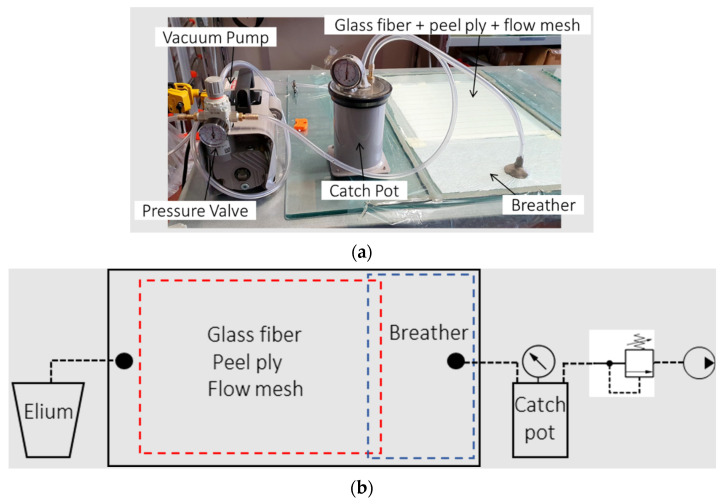
(**a**) Infusion setup and (**b**) representative sketch of the infusion setup.

Figure 1 shows the optimised infusion setup of the VARTM process executed in the Politecnico di Torino laboratory. As will be shown, this proposed setup is suitable for the acquisition of composite laminates without visible defects. The laminates were infused at room temperatures between 21 and 23 °C. A glass plate was used as a bottom mould. The infusion area was delimited by a butyl sealant tape, which ensures the sealing of the moulding area. The glass surface was treated with a releasing wax. Nine layers of glass fibre 2 × 2 twill fabric with a thickness of 0.3 mm each were placed over the glass mould and the weft fibres were aligned. A peel ply and flow mesh fabric were placed over the nine layers of glass fibres. The flow mesh ensures a proper and uniform flow of the resin during the infusion process, while the peel ply allows for a fast debonding of the final laminate from the upper layer. A breather layer was placed before the resin outlet, as shown in Figure 1a. The size of the breather was 200 × 350 mm for the infusion of a 350 × 350 mm laminate. Breather cloth is a non-woven polyester fabric that is designed to allow airflow throughout the vacuum bagging process. However, the breather mesh is very fine compared to the flow mesh; thus, it can slow the resin. In this case, the breather layer has a resin-break function: once the resin flow reaches the breather, it slows down and ensures a uniform resin distribution over the fibre layers. Once all the glass fibres were completely wetted by the resin, the resin outlet was closed. After 3 min, the resin outlet was closed to let the resin consolidate within the bag. The catch-pot was connected to the resin outlet by a silicone tube to prevent the resin from reaching the pump. Between the catch-pot and the vacuum pump, a pressure-regulating valve was placed to tune the infusion pressure.

The curing of the resin occurred at room temperature for 24 h. Then, the laminates were post-cured in the oven for one hour at 80 °C.

A 3 × 3 fully crossed design of experiment (DOE) plan was executed to evaluate the influence of the vacuum infusion pressure (VIP). The studied VIP values were 0.5, 0.7, and 0.8 bar and the studied degassing pressure values were 0.0, 0.85, and 1.0 bar. Accordingly, nine plates were produced using different parameter combinations. The degassing was executed after mixing the resin with the BPO hardener in a vacuum chamber for 4 min, according to the DOE plan. A maximum VIP of 0.8 bar was set since it was visually observed that at higher vacuum pressures the resin starts to boil, although the leak test was passed. Indeed, the overheating due to the exothermal reaction of free radical polymerisation together with the vacuum pressure could lead to the resin boiling [36,37,38]. This effect was evident at a vacuum pressure of 0.9 bar and, for this reason, the maximum VIP was set at 0.8 bar.

### 2.3. Experimental Tests

#### 2.3.1. Mechanical Tests

A material characterisation campaign was carried out to evaluate the mechanical response as a function of the process parameters. Each produced plate was cut by waterjet to obtain 5 tensile specimens, 5 bending specimens, 3 impact specimens, and 6 small samples to be used for density and void content measurements. The density measurement was performed using a helium gas pycnometer Anton Paar, Ultrapic 5000 (Graz, Austria).

The tensile tests were carried out on rectangular specimens having a length of 250 mm, a width of 25 mm, and a thickness from 1.48 mm to 1.62 mm (depending on the VIP used) according to the ASTM D3039 standard. The INSTRON 8801 (Norwood, CO, USA) servo-hydraulic universal testing machine was used to test the specimens in tension. The strain gauges were placed on the central section of the specimens and strain signals were acquired with a NI 9237 (National Instruments) acquisition board (Austin, TX, USA). Load signals were acquired using the testing machine standard 100 kN load cell, with a sampling rate synchronised with the strain data acquisition. The three-point bending tests were carried out according to the ASTM D790 standard, using a Zwick Roell (Ulm, Germany) electro-actuated testing machine equipped with a 5 kN load cell. The specimens had a length of 80 mm, a width of 12.7 mm, and a thickness from 1.48 to 1.62 mm (depending on the VIP used). The flexural strain and stress were obtained with the equations ε = 6Dd/L^2^ and σ = 3PL/(2bd)^2^, respectively, where P is the load, L is the support span, b is the width of the specimen, d is the thickness of the specimen, and D is the maximum deflection at the centre of the beam, according to the ASTM D790 standard and beam theory. The support span was computed by multiplying the average thickness by 16 as suggested by the standard.

Impact tests were performed to characterise the dynamic response of the specimens. The tests were performed using the Instron FractoVIS (Norwood, CO, USA) free-fall drop-dart testing machine following the ASTM D5628 standard. To evaluate the out-of-plane properties of the material, the specimens were clamped on a fixture along a circumference with a ring that presents an internal diameter of 76.2 mm. This ring is activated by compressed air at 8 bar. Square specimens, 100 × 100 mm, were used for the testing activity. The specimens had a thickness from 1.48 to 1.62 mm depending on the VIP used. 

The impacting energy is adjusted by setting the falling height and the impacting mass. The dart has a cylindrical shape, with a hemispherical tip having a diameter of 20 mm. The dart impacted exactly in the centre of the specimen. A falling mass of 10.89 kg was used, and the falling height was set equal for all impact tests to evaluate the specimen response by providing 10 J of supplied energy. This value was selected after some preliminary tests to obtain a full specimen perforation. A piezoelectric load cell was placed at the top extremity of the dart and the load signal was acquired at a frequency of 1 MHz. During the downward travel of the falling mass, some potential energy can be lost due to friction; therefore, the energy balance has to include the non-conservative term *W_f_*, as illustrated in Equation (1):

(1) $$E_{0}=mgh=\frac{1}{2}v_{p}^{2}+W_{f}\to W_{f}=mgh-\frac{1}{2}v_{p}^{2}$$

where *m* is the falling mass, *g* is the gravity constant, *h* is the initial falling height, *W_f_* is the non-conservative work due to friction, and *v_p_* is the velocity measured by an electro-optical device (photocell) that captures the dart velocity at the exact impact instant. This device is used also to trigger the load acquisition. The displacement of the dart during the impact is evaluated by double integrating the acceleration, retrieved by Newton’s law:

$$a(t)=g-\frac{F(t)}{m}$$

$$v\left(t\right)=\int_{t_{0}}^{t_{f}}a\left(t\right)dt+v_{p}$$

$$s\left(t\right)=\int_{t_{0}}^{t_{f}}v\left(t\right)dt$$

where *F*(*t*) is the time history of the force signal acquired by the load cell at the time *t*, *t_f_* and *t*_0_, respectively, are the last and the first time instant of the impact, and *v*(*t*) and *s*(*t*), respectively, are the velocity and the displacement of the dart calculated at the time *t*.

The energy *E_ab_* absorbed or dissipated by the specimen during the impact corresponds to the area under the force–displacement curve:

(2) $$E_{ab}=\int_{s_{0}}^{s_{f}}F\left(d\right)ds$$

where *s_f_* and *s*_0_ are the dart positions evaluated at *t_f_* and *t*_0_, respectively.

#### 2.3.2. Computed Tomography

Computed tomography (CT) analyses were carried out on the flexural specimens prepared with the three different VIP values (0.5, 0.7, and 0.8 bar). A voltage of 100 kV and a current of 100 µA were used for the analysis. The distance between the emission source and the detector was 1400 mm and the distance between the source and the object was set at 130 mm. The obtained resolution with these parameters was 18 µm. VG Studio Max (Hexagon, Stockholm, Sweden) was used to process the sequence of acquired CT. The Porosity/Inclusion analysis tool was used for the computation of the porosity within the composite specimens. This analysis confirmed the FVF values, which were respectively 0.50%, 0.51%, and 0.53% by infusion with a VIP of 0.5, 0.7, and 0.8 bar. The CT were carried out in J-Tech@PoliTO lab with a custom-built tomograph fabricated by Fraunhofer IKTS (Hermsdorf, Germany).

## 3. Results and Discussion

### 3.1. Process

**Figure 2:**
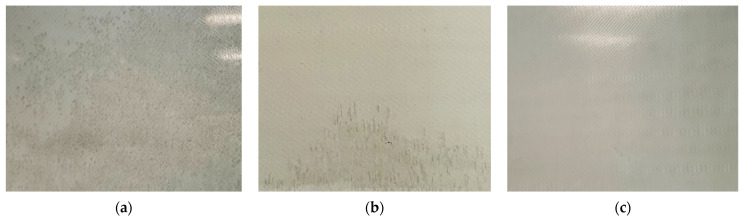
(**a**) Laminate obtained without breather; (**b**) laminate with VIP close to 1 bar; (**c**) laminate with VIP at 0.8 bar and breather before the outlet valve.

A proper and optimised infusion setup was needed to guarantee a good quality of laminates without either visible defects (voids, stripes, delamination) or being detectable using micrography. The breather dimensions were found to highly influence the visual defects within the laminate. Figure 2 shows the pictures of three plates processed with three different setups. Figure 2a shows a plate produced without the breather fabric. The consistent void and air spot content is due to the high flow front speed and the low viscosity of the resin. The low viscosity of the resin reduces friction so that it flows faster. However, a high flow front speed prevents the resin from properly wetting all the fibres and depositing homogeneously along the thickness of the sheets. This causes the air bubbles to remain trapped and not flow towards the outlet. Accordingly, as shown in Section 2.1, a breather fabric was placed before the resin outlet. The flow front slows down once it reaches the breather due to its low permeability, allowing the backward resin to homogeneously wet the glass fibres and bundles, letting the air bubbles flow towards the resin outlet.

However, the use of the breather alone only partially solved the problem. As highlighted in Section 2.1, a VIP close to 1 bar generated airstrips close to the resin outlet (Figure 2b). These strips are essentially air bubbles stretched along the flow direction, caused by cavitation. Figure 2c shows a laminate produced with a VIP of 0.8 bar. Neither air strips nor voids are evident. As reported in Section 2.1, after the glass fibres were wetted by the resin, the resin outlet was closed and, after 3 min, the resin inlet was closed. This helps the resin to be consolidated within the bag.

**Figure 3:**
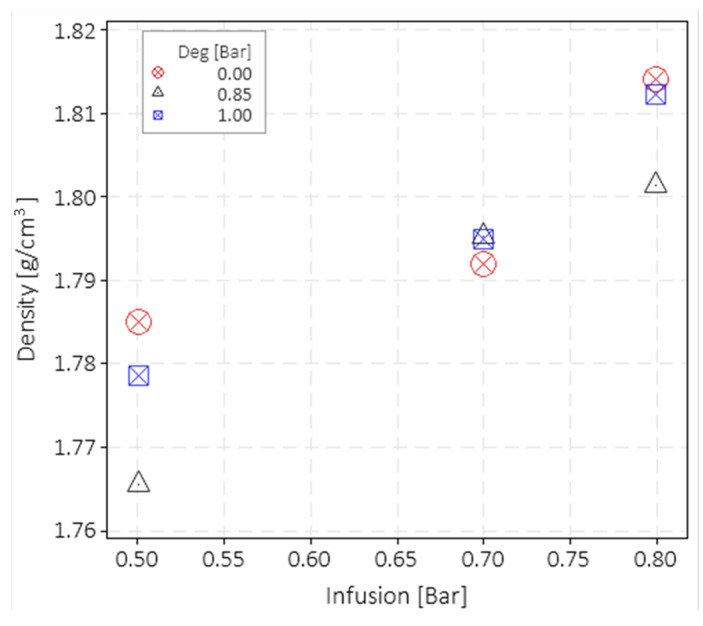
Measured density of the fabricated composite laminates.

It is worth noticing that the VIP value highly influences the final laminate thickness and density. Thicknesses of 1.62 mm (dev. St. 0.07 mm), 1.54 mm (dev. St. 0.015 mm), and 1.48 mm (dev. St. 0.023 mm) were obtained with a VIP of 0.5, 0.7, and 0.8 bar, respectively. Figure 3 shows the density measurement results obtained by the gas pycnometer as a function of the VIP and grouped by the degassing level.

A net increase in the density was found by increasing the VIP, which is a consequence of the laminate thickness reduction which, in turn, is caused by the reduction in the matrix volume fraction. This result is expected since an increase in pressure results in lower resin content. The density value dispersion for the same infusion level is not statistically significant, as confirmed by the ANOVA analysis. The ANOVA analysis was carried out with Minitab software. It can be stated that the degassing value does not influence the density with a confidence interval of 95%.

### 3.2. Mechanical Test Results

#### 3.2.1. Tensile Test

**Figure 4:**
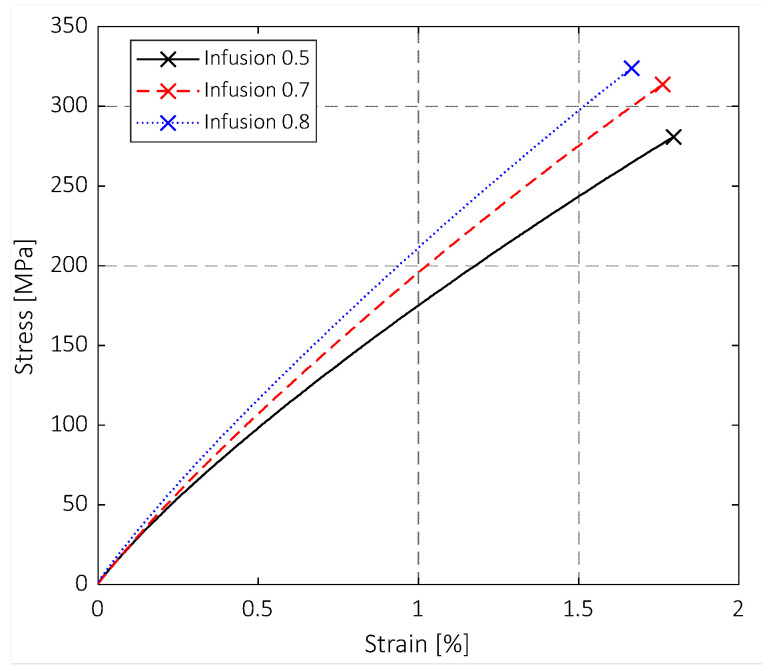
Tensile tests: stress–strain curves at different VIP.

**Figure 5:**
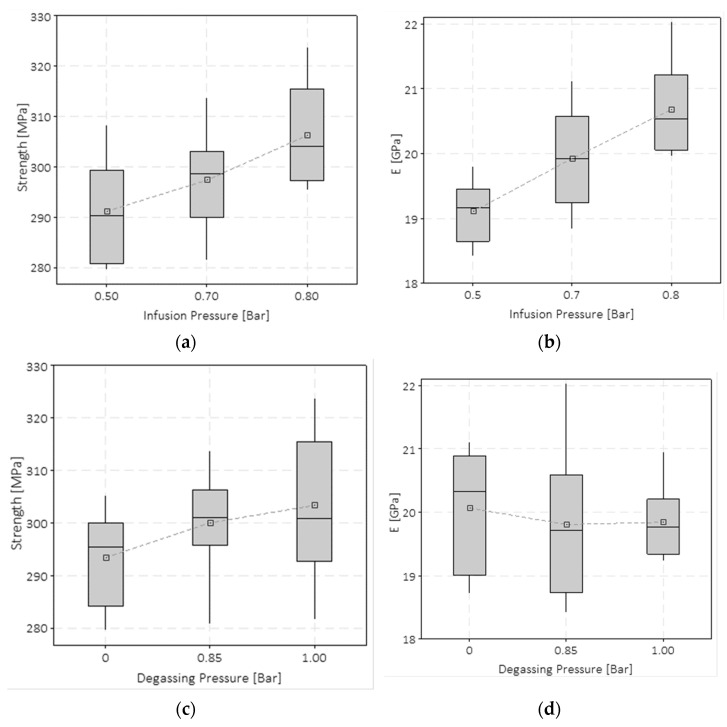
Summary of the tensile test results: (**a**) dependency of the maximum strength on the VIP; (**b**) Young’s modulus on VIP; (**c**) maximum strength on the degassing pressure; (**d**) Young’s modulus on the degassing pressure.

The results of the quasi-static tensile tests are shown in Figure 4 and Figure 5. The specimens produced with the highest VIP present the highest fibre volume fraction (FVF), and thus their performance is more dominated by the fibre characteristics than the specimens produced with the lowest VIP. Indeed, the FVFs for a VIP of 0.5, 0.7, and 0.8 bar are 0.50%, 0.51%, and 0.53%, respectively. This effect can be appreciated in Figure 4, which shows the stress–strain curves obtained from the tensile tests. Note that only three representative curves are drawn for the sake of clarity and data readability, but they well represent the population. The blue curve (VIP 0.8 bar) has a more fibre-dominant behaviour, and a nearly linear trend is visible up to the failure. In contrast, the black curve (VIP 0.5 bar), after the first part, drifts considerably from linearity. This trend is also related to the mechanical properties of the methylmethacrylate polymer [39]. This trend can be related to the matrix influence. Furthermore, it is worth noticing that lowering the VIP level emphasises the plastic behaviour of the coupons, and the strain to failure becomes higher, although the increase is not significant. This behaviour could be due to the increase in FVF, which influences the whole mechanical behaviour of the composite laminates. Thus, the mechanical behaviour is closer to that of glass fibres that present a fragile behaviour. Intermediate characteristics were found for specimens with a VIP of 0.7 bar.

ANOVA analysis showed that the VIP influences the tensile properties of both strength and elastic moduli. In the boxplot in Figure 5a, this trend is illustrated. The difference in strength from VIP 0.5 bar to VIP 0.8 bar was around 5%. It is noticeable that each of the boxes in Figure 5a includes a batch of tensile specimens tested for one specific VIP but prepared with different degassing pressure levels. Although a slight increase in the strength as a function of the degassing pressure can be appreciated in Figure 5c, the ANOVA analysis conducted with a significance level of 2.5% showed that both the degassing pressure and the interaction of the degassing pressure with the VIP did not have a statistically significant impact on the strength. Thus, grouping results according only to the infusion pressure level can be considered a meaningful data visualisation. The *p*-value for the VIP was 0.005; the *p*-value for the degassing pressure was 0.053; and that for the mixed term was 0.049. The same considerations can be made for Figure 5b, where the Young’s modulus is reported as a function of the VIP. In this case, the total increment of the Young’s modulus changing from VIP 0.5 to VIP 0.8 modulus is equal to 8.2%. The ANOVA conducted on the Young’s modulus variable showed a *p*-value of 0.011, 0.783, and 0.296, respectively, for the VIP, degassing pressure, and mixed term factors. Furthermore, in this case, the degassing pressure does not affect the response; it is even more clear in the boxplot in Figure 5d that no mean value trend is appreciable while high data dispersion is noticeable.

#### 3.2.2. Flexural Tests

**Figure 6:**
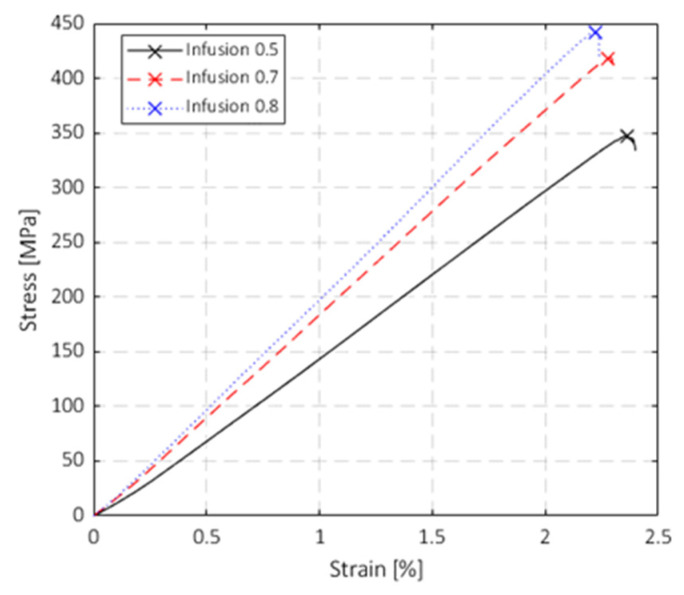
Flexural tests: stress–strain curves at different vacuum infusions.

**Figure 7:**
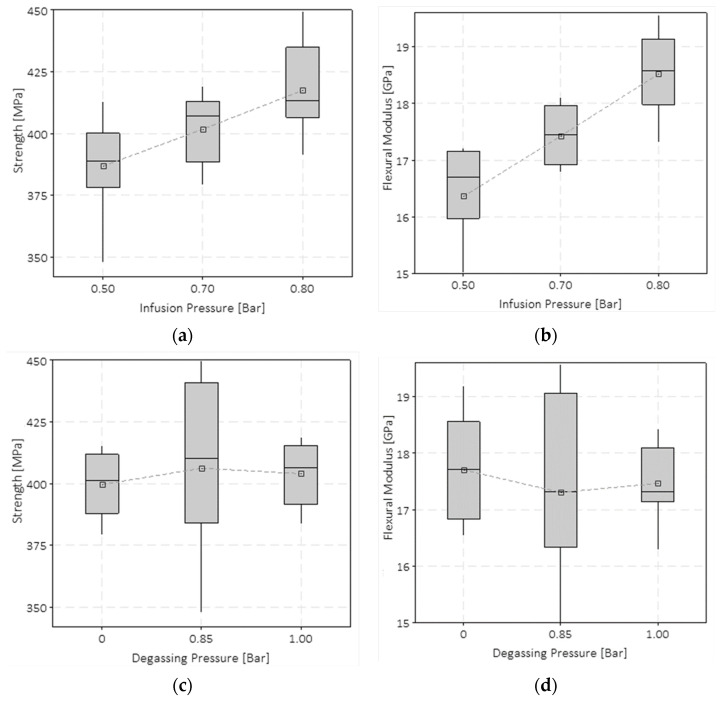
Summary of the flexural tests: (**a**) dependency of the maximum stress on the VIP; (**b**) dependency of the flexural modulus on the VIP; (**c**) dependency of the maximum stress on the degassing pressure; (**d**) dependency of the flexural modulus on the degassing pressure.

Figure 6 and Figure 7 show the results of the quasi-static three-point flexural tests carried out on standard specimens. The stress–strain curves at different VIPs are shown in Figure 6 (note that only three representative curves are drawn for sake of clarity and data readability). Differently from the tensile tests, all the tested specimens showed a nearly perfect linear behaviour until the failure. The brittle failure was chiefly caused by fibre rupture; after the first load drop, almost all the specimens kept their integrity, and no coupon half-splitting occurred.

Figure 7 shows that the blue curve (VIP 0.8 bar) presents the highest stress and the lowest strain at failure, while the black curve (VIP 0.5 bar) presents the lowest stress and the highest strain at failure. Similarly to the tensile tests, the mechanical behaviour of the composite laminates is more influenced by the fibre behaviour due to the higher FVF, which led to lower strain at break. In this case, the degassing pressure does not produce a direct effect on the material properties (Figure 7c,d). The reasons are again related to the fibre-dominant behaviour of the composite laminates prepared with higher VIP.

The bending response of the material is more sensitive to the variation of the production process parameters. The increasing trends of the flexural maximum strength and the flexural modulus are more evident, and the data dispersion within each VIP group is lower compared to the tensile test case. The variations of the strength and the modulus from VIP 0.5 to 0.8 bar were 7.5% and 14%, respectively. For all the considered VIPs, the flexural modulus was slightly lower than the tensile modulus, even if all the samples demonstrated higher strength. The dependencies of the maximum flexural strength and flexural modulus on the VIP are shown in Figure 7a and Figure 7b, respectively. Figure 7c,d show the dependencies of the flexural strength and modulus on the degassing pressure, respectively. In this case, degassing before infusing does not influence the results.

#### 3.2.3. Impact Drop-Dart Tests

**Figure 8:**
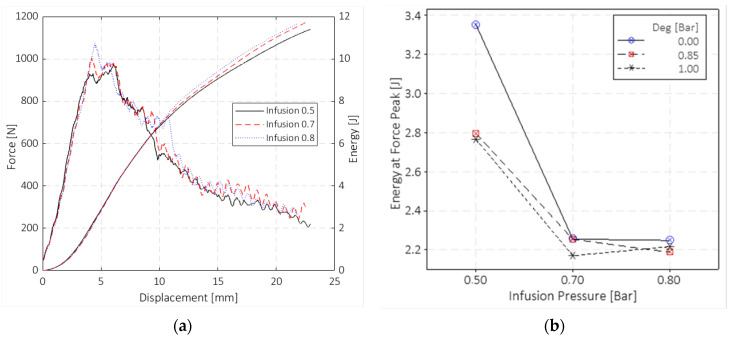
Summary of the drop-dart impact tests: (**a**) force–displacement and energy–displacement curves; (**b**) energies at peak forces.

The impact response of the materials was evaluated by carrying out impact tests, according to the ASTM D5628 standard, at 10 J. These test results showed a non-clear dependency on the vacuum infusion and degassing pressures, differing from the ones obtained for the tensile and flexural tests. Neither the degassing pressure or the VIP significantly affect the peak loads and absorbing energy capability of the plates subject to impact. Figure 8a shows through a double *y*-axis graph the results of the impact tests in terms of force–displacement and energy–displacement curves. Note that although only three curves are shown for data readability, these trends are representative of all the tested samples with the same VIP level. The data were not grouped as a function of the degassing pressure level as it gave no effect on the impact response. The test data were filtered with a Butterworth filter to slightly smooth the signal noise.

A slight increase in the peak force can be appreciated with the VIP increment. It is worth noticing that for almost all the VIP 0.5 bar samples, the peak force was shifted towards higher displacements for VIP 0.7 and 0.8 bar specimens. As a consequence, the energy at the peak force for the VIP 0.5 bar samples was around 25% higher than that for the VIP 0.7 and 0.8 bar specimens (Figure 8b). Figure 8b shows the trend of the absorbed energy at the force peak as a function of the two pressure variables. The energy at peak force gives an important indication of the energy level at which the main failure occurs and the material loses its carrying capability. It can be noted that the degassing pressure does not significantly affect the impact behaviour, except for the case of the 0.0 bar degassing pressure and 0.5 bar VIP, whereas the VIP value has a significant influence. For the VIP 0.5 bar cases, the absorbed energy at the force peak has higher values due to the higher resin content, and the glass layers are less compact due to the lower infusion pressure. This explains why the delamination propagated more widely, leading to the main failure (due to fibre rupture) occurring at greater deformations.

#### 3.2.4. Fracture Surfaces and Microscopy Analysis

**Figure 9:**
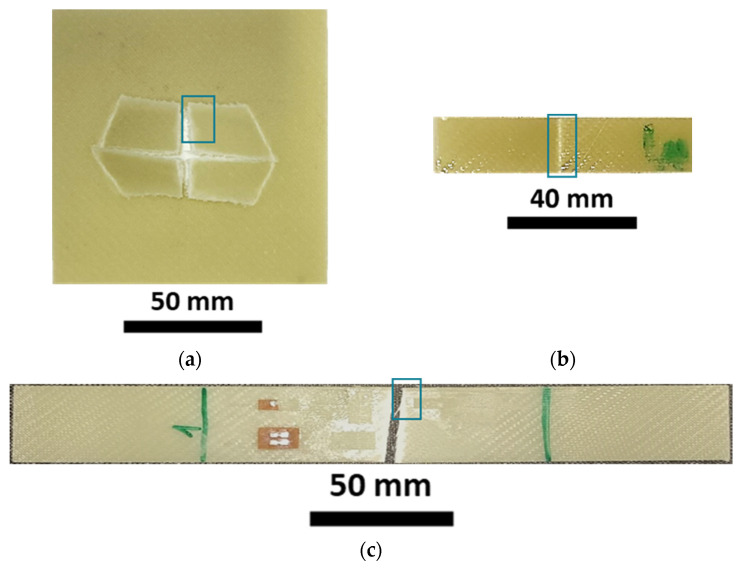
Representative failure surface obtained in impact tests (**a**), flexural tests (**b**), and tensile tests (**c**).

The fracture surfaces of the impact, flexural, and tensile specimens are shown in Figure 9a, Figure 9b and Figure 9c, respectively. Figure 9a illustrates that the material fractured generating four fronds which, however, remained attached to the specimen and did not generate debris. During the dart penetration, the fronds plastically deformed during bending, reaching angles of almost 70 degrees with respect to the plane of the specimen. Figure 9b,c show representative flexural and tensile specimens after the test. The specimens experienced ruptures in the middle according to the adopted standards.

**Figure 10:**
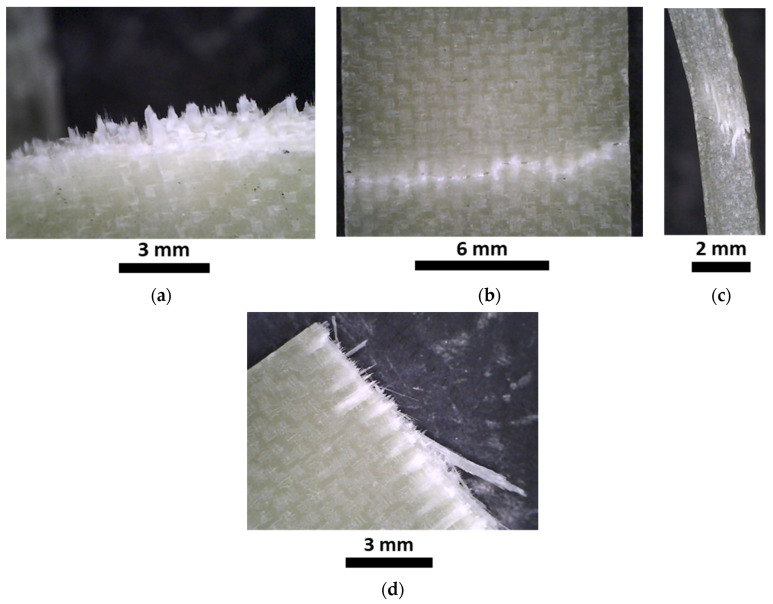
Magnification of the composite fractures related to impact specimen (**a**); lower side (tension) of the flexural specimen (**b**) and its section (**c**); tensile specimen (**d**).

Figure 10 shows the magnification of a small area related to the fracture surfaces. The reported areas are those depicted in Figure 9a–c. Figure 10a shows a representative failure surface of the impact specimens. The out-of-plane deformations on the specimen trigger the fibre breakage on the tension side (visible because of the pulled-out fibres in the lower part of the specimens). Figure 10b,c show the failure surface of the flexural specimens: the lower side of the specimen and the side section, respectively. The lower side of the specimen, opposite to the pin that is transmitting the load, is again the tension side. It is possible to see that the flexural specimens did not experience a complete failure but only a fibre breakage on the tension side, as shown in the right side of Figure 10c. Finally, Figure 10d shows a magnification of the failure surface related to tensile tests. This last case presents a similar failure surface with some visible pulled-out fibre.

**Figure 11:**
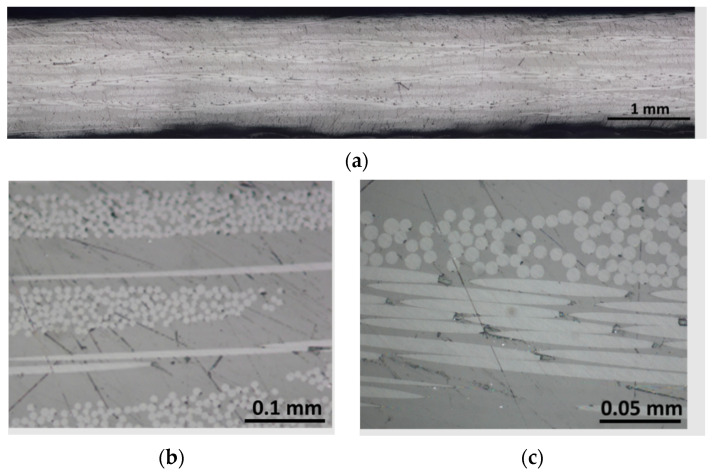
Micrography of the plate cross sections at three different magnifications (**a**–**c**).

Finally, Figure 11 shows that the proposed infusion process methodology can fabricate GFRP laminates free of visible defects at three different VIPs of 0.5, 0.7, and 0.8 bar, and with low void contents as shown by the CT analysis in Section 3.2.5. Figure 11a–c report different magnifications with three different scales, of 1, 0.1, and 0.05 mm, respectively, of GFRP prepared with a vacuum infusion of 0.5 bar; however, this is representative of all the different laminates since no significant differences were observed using the microscope. Many works in the literature showed that Elium resin cannot be infused with the VARTM technique without load contents. For example, Bhudolia et al. [33] found a void content of 6% for laminates infused at 0.5 bar. As can be appreciated from the micrographs in Figure 11, the cross-section of the plates appears to be free of perceptible voids or defects, and the resin is well-distributed around the fibres oriented both in the warp and weft directions. The higher magnifications shown in Figure 11b,c show that there are no voids in the resin (darker surface in the images). Figure 11b shows the longitudinal and transverse tows of the glass fibres. The transverse ones are visible due to the circular section of the fibres, while the longitudinal ones are recognisable due to the longer continuous fibres.

#### 3.2.5. Micro-CT Analysis

**Figure 12:**
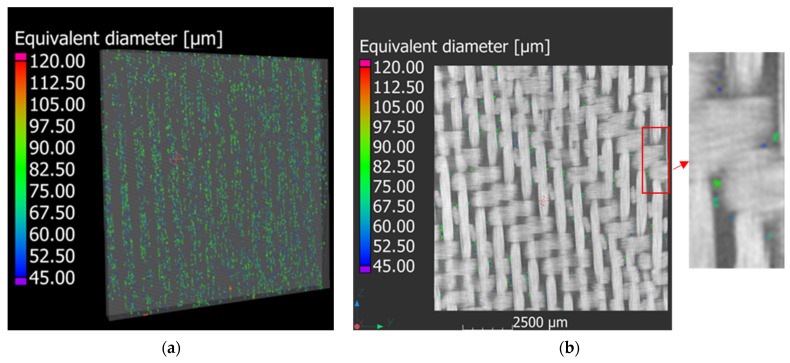
µCT analysis of the specimens: (**a**) 3D view of the reconstructed CT analysis; (**b**) location of the voids in 2D images and a magnification.

Figure 12a shows a reconstructed volume of the images acquired with the CT scans. Figure 12a illustrates that the voids follow specific lines oriented along the vacuum infusion direction. Although the volume element presented in Figure 12a can provide a general overview of the whole presence of voids, it is not able to provide the exact location. For this reason, Figure 12b is presented to show that the voids are located between the crossed roving. This behaviour was also reported in [40]. The Porosity module of VG studio Max was used to determine the void content of the three composite materials used with the three different adopted VIPs, and a void content of 1.17%, 1.12%, and 0.74% was found for the composite materials prepared with a VIP of 0.5, 0.7 and 0.8 bar, respectively. However, Mehdikhani et al. [40] and Protz et al. [41] showed that the tensile and compressive behaviour of glass-fibre-reinforced polymers produced with the VARTM methodology is not affected if the void content is below 5%. Finally, Bhudolia et al. [33] showed that a good level of voids between 0.88% and 1% was obtained using an infusion pressure of 0.5 bar and a subsequent consolidation at 0.33 bar for thin and thick laminates. Thus, the optimised infusion methodology and parameters designed in the present work enable the production of GFRP laminates at a consolidation pressure of up to 0.8 bar instead of 0.33. Contrarily, Bhudolia et at. [33] illustrated that, by using a vacuum infusion of 0.5 bar, void contents between 4 and 8% were achieved for thin and thick laminates.

## 4. Conclusions

The mechanical properties of GFRP manufactured with Elium matrix, a special type of thermoplastic polymer, were evaluated in this work as a function of the two main process parameters, the vacuum infusion and degassing pressures. Further, a methodology for the infusion of composite laminates at vacuum pressures higher than 0.5 was proposed. This infusion VARTM methodology differs from the one reported in the literature [33], which allows the production of laminates with void content of 0.88% at a vacuum pressure of 0.5 bar and consolidation pressure of 0.33 bar. The methodology illustrated in the present work can produce laminates with low voids content, of 0.74–1.14%, at a vacuum infusion pressure between 0.5 and 0.8 bar.

The mechanical tests showed that the vacuum infusion pressure (VIP) significantly affects Young’s modulus, tensile strength, flexural modulus, and flexural strength. Tensile tests showed that an increase in the infusion pressure from 0.5 to 0.8 bar led to an increase in the strength of ~6% and Young’s modulus of ~10%. Flexural tests showed that when the VIP increases from 0.5 to 0.8 bar, the increment of the flexural strength and elastic modulus is ~8% and ~15%, respectively. The results of the drop-dart impact tests do not show a significant effect of the considered degassing pressure and VIP values on the peak force values. An increased amount of the absorbed energy at the peak force was found for the composite plates infused at the vacuum pressure of 0.5 bar. Although tensile, flexural, and impact properties were analysed, this work is intended to be a preliminary analysis to evaluate the effect of the process parameters concerning some of the most used mechanical standard tests.

## Figures and Tables

**Table 1:** Elium^®^ technical data.

Brookfield Viscosity (25 °C)	mPa	100
Liquid Density	g/cm^3^	1.01
Gel time (25°)	min	60–90
Peroxide Ratio (BPO)	Wt%	3
Tensile Strength	MPa	56
Tensile Modulus	GPa	2.6
Elongation at break	%	3.44

## Data Availability

Data are available on request.
